# Algometry with a clothes peg compared to an electronic pressure algometer: a randomized cross-sectional study in pain patients

**DOI:** 10.1186/1471-2474-12-174

**Published:** 2011-07-25

**Authors:** Niklaus Egloff, Nicole Klingler, Roland von Känel, Rafael JA Cámara, Michele Curatolo, Barbara Wegmann, Elizabeth Marti, Marie-Louise Gander Ferrari

**Affiliations:** 1 Department of General Internal Medicine, Division of Psychosomatic Medicine, Inselspital, Bern University Hospital, CH-3010 Bern, Switzerland; 2 Department of Anaesthesiology and Pain Therapy, Inselspital, Bern University Hospital, CH-3010 Bern, Switzerland; 3 Department of General Internal Medicine and Clinic for Orthopaedics, Inselspital, Bern University Hospital, CH-3010 Bern, Switzerland

## Abstract

**Background:**

Hypersensitivity of the central nervous system is widely present in pain patients and recognized as one of the determinants of chronic pain and disability. Electronic pressure algometry is often used to explore aspects of central hypersensitivity. We hypothesized that a simple pain provocation test with a clothes peg provides information on pain sensitivity that compares meaningfully to that obtained by a well-established electronic pressure algometer. "Clinically meaningful" was defined as a medium (r = 0.3-0.5) or high (r > 0.5) correlation coefficient according to Cohen's conventions.

**Methods:**

We tested 157 in-patients with different pain types. A calibrated clothes peg was applied for 10 seconds and patients rated the pain intensity on a 0 to 10 numerical rating scale. Pressure pain detection threshold (PPdt) and pressure pain tolerance threshold (PPtt) were measured with a standard electronic algometer. Both methods were performed on both middle fingers and ear lobes. In a subgroup of 47 patients repeatability (test-retest reliability) was calculated.

**Results:**

Clothes peg values correlated with PPdt values for finger testing with r = -0.54 and for earlobe testing with r = -0.55 (all p-values < 0.001). Clothes peg values also correlated with PPtt values for finger testing with r = -0.55 (p < 0.001). Test-retest reliability (repeatability) showed equally stable results for clothes peg algometry and the electronic algometer (all r-values > 0.89, all p-values < 0.001).

**Conclusions:**

Information on pain sensitivity provided by a calibrated clothes peg and an established algometer correlate at a clinically meaningful level.

## 1. Background

Quantification of the human painful sensory experience is essential for diagnostic and pain monitoring purposes. In recent years, more advanced techniques have been developed for induction and assessment of pain. Different pain modalities (thermal, electrical, chemical and mechanical) are applied [1] of which the measurement of *mechanical *pain sensitivity is the most widely used in pain research. Many clinical centres routinely measure *pressure pain sensitivity *while assessing pain patients. *Pressure pain detection threshold *(PPdt) is defined as the point in which a steadily increasing non-painful pressure stimulus turns into a painful pressure sensation. *Pressure pain tolerance threshold *(PPtt) is defined as the highest level of pain, which a subject is prepared to tolerate [2]. A *reduced *PPtd is a sign of allodynia or hyperalgesia. The diagnostic identification of such pain perception abnormalities has a major impact on the patient's comprehension of the pain and the pain therapy [3]. Central hypersensitivity for pain has been detected in *fibromyalgia *[4] but is also present in many of the so-called *somatoform *or *functional pain syndromes *[5].

Typically, the assessment of mechanical pain sensitivity is performed by means of an electronic pressure algometer. By applying measured amounts of pressure the individual *pressure pain detection threshold *(PPdt) and/or *pressure pain tolerance threshold *(PPtt) are determined. This type of quantifiable application of mechanical pressure is considered as the gold standard for measuring pressure pain sensitivity [6]. However, the costs related to the equipment limit the use of electronic pressure algometry in everyday clinical practice.

Therefore, we aimed at developing and evaluating a simple and standardised pain provocation test: a physically calibrated clothes peg (UK) [respectively clothes pins (US)] acting as a standardized pain stimulus.

Our hypothesis was that this simple and easy-to-administer pain provocation test would provide information on pain sensitivity which correlates clinically meaningfully with the results provided by a well-established electronic pressure algometer. *Clinically meaningfully *was defined as a *medium *(r = 0.3-0.5) or *high *(r > 0.5) correlation coefficient according to Cohen's conventions [7].

## 2. Methods

### 2.1 Patients and Design

The study protocol was approved by the local ethics committee.

At the Bern University Hospital 170 consecutive in-patients suffering from pain were asked to participate, 157 of them consented to participate in the algometric study.

Our aim was to explore the two test methods in a wide range of pain types. Therefore, we recruited patients in different clinical departments, i.e. the orthopaedic department (*orthopaedic pain group*) and the medical-psychosomatic department (*medical-psychosomatic pain group*). Eligibility criterion was an acute or chronic pain condition as the primary clinical problem. Patients with pain disorders of *neuropathic *origin (e.g. peripheral neuropathy or radiation syndromes) were not included because of expected inhomogeneous peripheral pain sensitivity condition. We also excluded patients with craniocerebral trauma and those with an inflamed or traumatised algometric measuring site (e.g. infected ear lobe, broken hand).

In order to determine the correlation between the algometric methods, the tests were performed using a randomized cross-sectional design. Moreover, both tests were carried out during the same session. The order of the tests was individually determined by flipping a coin.

To compare test-retest reliability (repeatability) of the two methods, clothes peg test and pressure algometry were repeated in a consecutive subgroup of chronic patients of the medical-psychosomatic department with pain duration of more than 6 months. Patients needed to stay in the hospital for at least 7 days to qualify for repeatability analysis. The retest assessments were performed exactly the same way as the previous test session, i.e. at the same time of the day, under identical conditions, and by the same tester.

To assess the psychological status of the patients, they were asked to fill in a self-rating test each time they underwent pain testing. We applied the validated German Version of the depression subscale of the Hospital Anxiety and Depression Scale (HADS-D), which is designed to rate symptoms and levels of depression in non-psychiatric populations [8]. Total HADS-D scores (range 0-21) were interpreted either as clinically relevant or clinically not relevant according to a cut-off-value of ≥ 9 for clinically relevant depression [9].

### 2.2. Instrumentation and Application

**Figure 1:**
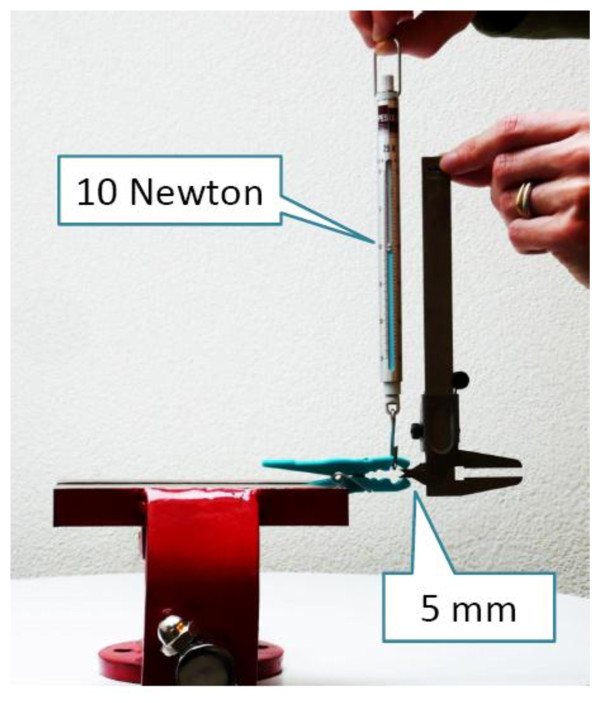
**Measurement of the Clamping Force of a Clothes Peg**. We selected clothes pegs in which the spread of 5 mm (controlled with a caliper) could be reached with a force of 10 Newton in vertical direction (controlled with a spring scale).

For our pain provocation test we used polypropylene clothes pegs (type MaxiMedium 2083, size 78 × 10 mm, imported 2004 by C.C. Hansen, Denmark, produced in Hangzhou, China). For algometric procedures we selected clothes pegs with a clamping force of exactly 10 Newton at an extension of 5 mm (Figure 1). Pilot testing showed stable measurement readings between 0 and +35 degrees centigrade. Repeated use (more than 2,000 consecutive tests) did not result in any decrease in clamping force. As the manufacturer of MaxiMedium in the meantime (2010) uses a thinner wire for this clothes peg type, we had to regalvanize the springs of the new series with nickel to obtain the original clamping force of 10 Newton (Algopeg, galvanized by Rolf Helbling, Biel, Switzerland).

A separate sample of clothes pegs with *lower *or *higher *clamping force (4, 6, 14 Newton, respectively) was selected for training purposes (see below).

For testing PPdt and PPtt, we used an established electronic pressure algometer with a probe of 1.0 cm^2 ^(Somedic Type II, size 161 × 170 × 30 mm, Somedic Production AB, Hörby, Sweden). The instrument was calibrated following the standard protocol as recommended by the manufacturer: A 1-kg weight provided by the manufacturer was applied to the 1.0 cm^2 ^diameter applicator head attached to the end of the nozzle. The acceptable calibration values ranged from 99 to 101 kPa.

Before algometry was started, the tester explained the procedure to the patients. The experiment was performed in a quiet setting without any interruptions and shielded from other patients. Each of the two tests was once performed for training purposes as a *trial run*. To avoid any local pain sensitization, the trial run tests were performed on the *index *finger, the subsequent actual testing was performed on the *middle *fingers. Additionally, the *trial run *was deliberately carried out with several "demo clothes pegs" *of different clamping forces *(calibrated for 4, 6 or 14 Newton). This preliminary challenge with clothes pegs of obviously *varying forces *was done with the intention to let the patients assume that test pegs can *objectively *cause different degrees of pain ("blinding"). The use of clothes pegs of varying forces was important especially with regard to the retest-setting. It follows that the patients could not automatically presume that every test peg causes *the same *pain intensity.

All actual *test series *were carried out with a 10 Newton-calibrated clothes peg. For each measurement the test pin was applied on the middle fingers and ear lobes for 10 seconds. The patient afterwards indicated the pain intensity on a numerical pain rating scale (NRS) on which 0 stands for „*no pain*" and 10 for „*the most intense pain imaginable"*. Since pain increases during the 10 seconds stimulation, patients were explicitly asked about the intensity of pain they perceived at the *end *of the test (i.e. at 10 seconds).

**Figure 2:**
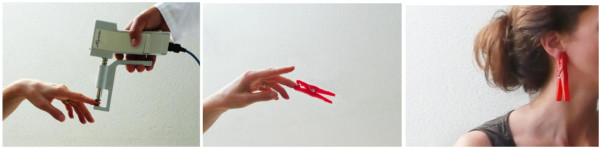
**The Two Compared Algometric Methods**. **a** shows the measurement of pressure pain sensitivity with an electronic algometer. **b **and **c **show the pain provocation test with a calibrated clothes peg.

The tests were performed on both sides of the body to average out possible side-specific differences in perception [10]. According to the protocol, both instruments were applied successively to the right middle finger, the left middle finger, the right ear lobe and the left ear lobe. The electronic algometer and the clothes peg were applied to the nails of the middle fingers without touching the nail fold (Figures 2a and 2b). Pain tests on the ear lobes were performed on the central soft tissue part without touching the ear cartilage (Figure 2c).

Each of the four anatomical sites was tested once with the clothes peg. Electronic algometry for testing the *pressure pain detection threshold *(PPdt) was performed 3 times on each site and the average was used for data analyses. The electronic algometer was set to deliver a steadily increasing pressure (50 kPa for one second). The patients pressed the button as soon as the pressure sensation turned into pain. At this time, the algometer freezes the pressure, its value can be read from the display. A 1 min break between each test procedure was planned in order to avoid local pain sensitization.

At the end of the clothes peg and PPdt test series a one-time determination of the *pressure pain tolerance threshold *(PPtt) was carried out by applying the electronic algometer to the middle finger of the dominant hand. For this purpose, the applied pressure was increased in steps of 50 kPa. The patient pressed the button as soon as his or her maximally tolerable pain level was attained. Considering that this test is particularly unpleasant, we performed it only once.

The whole test series was repeated in a subgroup of long-time patients for the determination of *test-retest reliability*

### 2.3 Data Analysis

For the analyses we used SPSS 17 for Windows. Variables were expressed as percentages, *mean values *with standard deviation (SD) and because some variables showed a skewed distribution, also as *median *plus interquartile range (IQR). The level of significance was set at p < 0.05 (two-tailed). As the data were not normally distributed, correlation with Spearman's rho was calculated to measure the correlation between the electronic and clothes peg algometric method. According to Cohen's conventions, r = 0.3-0.5 corresponds to a correlation size of a medium effect and r > 0.5 corresponds to a large effect [7].

Repeatability coefficients and plotted test-retest differences against test-retest means using the Bland Altman technique were computed for those patients who qualified for repeatability analysis (cf. chapter "Patients and design") [11].

In addition, we plotted test-retest differences against test-retest time in order to investigate whether repeatability was time-dependent.

## 3. Results

### 3.1 Patient Characteristics

The PPdt and clothes peg tests could be performed in all of the 157 patients. Six patients refused to test PPtt because of fear of severe pain.

Main characteristics of the patients are shown in Table 1. Seventy (45%) patients were recruited from the surgical orthopaedic department and 87 patients (55%) from the medical-psychosomatic department. In both departments the treating physicians made diagnoses according to the usual standards and classification systems (ICD-10). Twenty-six patients showed more than one diagnosis (20% of the orthopaedic patients, 13% of the medical-psychosomatic patients).

**Table 1:** Characteristics of Patients

Variable	All patients (n = 157)	Orthopaedic patients (n = 70)	medical-psychosomatic patients (n = 87)	Patients retested (n = 47)
Female sex	51%	41%	55%	62%

Age (years) *	52.4 (15.3) 51 (43-62)	57.8 (17.4) 59 (44-74)	48.0 (11.7) 49 (42-55)	47.1 (12.1) 48 (42-54)

Baseline pain (NAS score) *	5.0 (2.3) 5 (3-7)	3.6 (1.4) 4 (2-4)	6.1 (2.4) 6 (4-8)	6.1 (2.5) 7 (4-8)

Acute pain, < 6 months	24%	46%	7%	7%

Chronic pain > 6 months	76%	54%	93%	93%

Mean duration of pain (months) *	62.1 (98.7) 24 (6-60)	18.1 (27.1) 6 (1-24)	97.4 (119.4) 48 (19-96)	77.6 (94.5) 42 (18-87)

Monolocular pain	43%	70%	21%	18%

Multilocular pain	57%	30%	78%	82%

Non-steroidal anti-inflammatory drugs	64%	87%	45%	47%

Opioid drugs	27%	13%	38%	29%

Antidepressant drugs	48%	7%	81%	84%

HADS-D Depression Score*	7.7 (5.1) 6 (3-11)	4.5 (2.8) 4 (2-6.8)	10.4 (5.1) 10 (5.3-14)	10.7 (5.2) 10 (4.8-14.3)

HADS-D = Depression subscale of the Hospital Anxiety and Depression Scale

*) values are given as *mean *(SD) and *median *(interquartile range)

We found a wide range of different pain types: Approximately half of the *orthopaedic *patients (n = 34) suffered from acute traumatic pain: 12 patients had a trauma of upper extremities or shoulders, 17 had bone fractures or joint lesions of the lower extremities, 9 had a trauma in the thoracic, spinal or pelvic region. Seven patients had a polytrauma with ≥ 3 lesions. The other half of the orthopaedic patients (n = 36) suffered from pain of osteoarthritic degenerative origin: 11 had hip arthritis, 11 had knee arthritis, 7 had degenerative shoulder problems, and 10 had degenerative low back pain. The vast majority of the patients of the *medical-psychosomatic *department suffered from chronic pain syndromes which were incompletely explained by persistent peripheral tissue damage: 5 patients suffered from chronic tension headache, 11 had chronic cervical pain syndromes, 3 suffered from chronic temporomandibular or atypical facial pain syndromes, 27 had chronic low or upper back pain, 8 had chronic abdominal or pelvic pain, 6 suffered from functional hemisided pain syndromes, and 16 had chronic atypical postsurgical pain syndromes. Twenty-three of the medical-psychosomatic patients suffered from pain without any structural findings or any history of peripherally induced pain: 15 of them were diagnosed with fibromyalgia, 8 with trunk accentuated somatoform pain syndrome.

Forty-one (47%) of the medical-psychosomatic pain patients showed clinically elevated depression scores (HADS-D ≥ 9 points), whereas only 6 patients (9%) had elevated depression-scores in the orthopaedic group.

**Figure 3:**
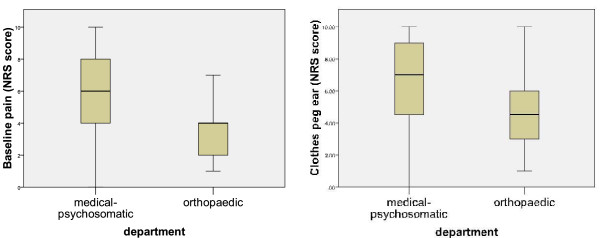
**Pain Characteristics of the Two Clinical Groups**. Our aim was to compare the two algometric test methods in a wide range of pain types. Therefore we recruited patients from the orthopaedic department and the medical-psychosomatic department. **a** illustrates the distribution of the baseline pain values (NRS) in both groups. **b **illustrates the distribution of the pain sensitivity values (NRS) of the ear lobe provoked by clothes pegs. The box-and-whisker-plots show the median with interquartile range (box: 25^th ^and 75^th ^percentile) and 5^th ^and 95th percentile (whiskers) of the data distribution.

Pain sensitivity data are described in Table 2. The distribution of baseline pain values and clothes peg data (ear lobe) are illustrated with box-and-whisker plots (Figure 3).

**Table 2:** Pain Sensitivity Data

Variable	All patients (n = 157)	Orthopaedic patients (n = 70)	Medical-psychosomatic patients (n = 87)	Patients retested (n = 47)	
				
				**1**^**st **^**measure**	**2**^**nd **^**measure**
finger PPdt (kPa)^1)^	197 (103) 188 (122-252)	214 (77) 205 (151-274)	183 (119) 152 (99-243)	164 (96) 145 (95-232)	165 (101) 159 (87-222)

ear PPdt (kPa)^2)^	147 (78) 137 (94-196)	154 (62) 142 (107-195)	142 (91) 128 (75-196)	116 (73) 109 (62-155)	129 (75) 123.2 (85-172)

finger PPtt (kPa)	376 (163) 348 (269-495)	366 (104) 344 (297-438)	384 (200) 359 (216-526)	323 (162) 329 (193-434)	311 (146) 312 (192-410)

clothespin finger (NRS score)^1)^	2.8 (2.5) 2.0 (1.0-4.0)	1.8 (1.3) 2.0 (1.0-2.0)	3.6 (2.9) 3.5 (1.0-5.5)	4.1 (2.7) 3.5 (2.0-6.0)	4.3 (3.0) 4.0 (2.0-7.5)

clothespin ear (NRS score)^2)^	5.6 (2.8) 5.0 (3.0-8.0)	4.4 (2.1) 4.5 (2.8-6.0)	6.6 (2.9) 7.0 (4.5-9.0)	6.9 (2.7) 7.5 (5.0-9.0)	6.9 (2.8) 7.5 (5.0-9.5)

NRS = Numerical pain rating scale; kPa = kilo Pascal; PPdt = Pain pressure detection threshold; PPtt = Pain pressure tolerance threshold

All values are given as *mean *(SD) and *median *(interquartile range)

^1) ^Mean value of left and right middle fingers

^2) ^Mean value of left and right ear lobes

### 3.2 Correlations Between the two Test Methods

The comparison between clothes peg values and PPdt (respectively PPtt) revealed an inverse correlation across all patients, i.e. the higher the pain in the clothes peg test, the lower the pressure pain threshold, and vice versa. Table 3 shows partial correlation coefficients for the associations between the two pain measurement methods. It can be seen that the absolute size of the correlation coefficients is similar in both patient groups and both anatomical test sites. According to Cohen's conventions only one value corresponds to a correlation size of a *medium *effect (r = 0.3-0.5), all other correspond to a *large *effect (> 0.5).

**Table 3:** Correlation of Electronic and Clothespin Algometric Methods

Correlation	All patients (n = 157)	Orthopaedic patients (n = 70)	medical-psychosomatic patients (n = 87)
PPdt finger *versus *Clothespin finger			
	*r = -0.54*	*r = -0.38*	*r = -0.59*
		*p = 0.002*	*p < 0.001*

PPdt ear *versus *Clothespin ear			
	*r = -0.55*	*r = -0.53*	*r = -0.51*
		*p < 0.001*	*p < 0.001*

PPtt finger *versus *Clothespin finger			
	*r = -0.55*	*r = -0.53*	*r = -0.62*
		*p < 0.001*	*p < 0.001*

PPdt = pressure pain detection thresholds; PPtt = pressure pain tolerance thresholds

Correlation with spearmans rho

### 3.3 Test-retest Reliability (Repeatability)

**Figure 4:**
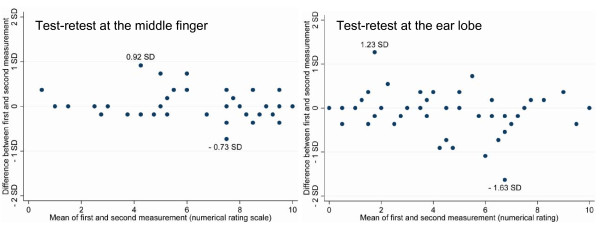
**Repeatability of Clothes Peg Algometry**. **a **illustrates the test-retest reliability of the clothes peg test at the *middle finger*. **b **illustrates the test-retest reliability of the clothes peg test at the *ear lobe*. Both plots reflect the statistical agreement between the two clinical measurements following the Bland Altman technique [11]. Test-retest difference was not related to pain intensity. The null line stands for identical test and retest values. According to the British Standards Institution, at least 95% of the differences between test and retest are expected to be within two standard deviations from the null line to assume good repeatability http://www.bsigroup.com. All methods of measurement fulfilled this criterion; 100% of the differences were within these limits (maximum and minimum distances from the null line are indicated). In addition, no linear relation between pain intensity and test-retest difference was visible.

**Figure 5:**
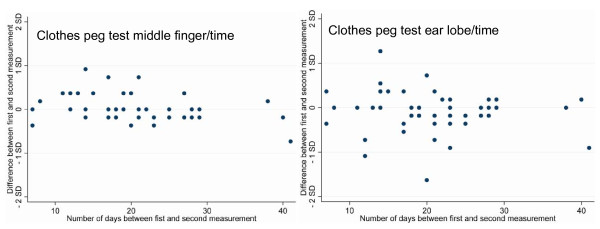
**Repeatability of Clothes Peg Algometry Controlled for the Time Between the Two Measurements**. **a b **Test-retest difference was neither related to pain intensity nor to time between test and retest. There is no linear relation between test-retest time and test-retest difference.

Forty-seven patients from the medical-psychosomatic department qualified for repeatability analysis. Test-retest time varied between 7 and 41 days (mean: 21; SD 8). For both algometric methods, repeatability coefficients were smaller than one SD of test-retest means, indicating a more than four-fold lower within-individual variance over time than between individuals (Table 4). Test-retest difference was neither related to pain intensity (Figure 4) nor to test-retest time (Figure 5).

**Table 4:** Repeatability Data

Method of measurement	Repeatability coefficient	Mean of the differences	Standard deviation of the means of 1^st ^and 2^nd ^measurements	Standardised repeatability coefficient	Standardised mean of the differences
PPdt finger (kPa)^1)^	89.7	- 1.44	96.3	0.93	- 0.01

PPdt ear (kPa)^2)^	54.2	- 10.5	72.3	0.75	- 0.15

Clothespin finger (NRS score)^1)^	2.64	- 0.25	2.76	0.96	- 0.09

Clothespin ear (NRS score)^2)^	1.72	0.02	2.72	0.63	< 0.01

NRS = Numerical pain rating scale; kPa = kilo Pascal; PPdt = Pain pressure detection thresholds

^1) ^Mean value of left and right middle finger

^2) ^Mean value of left and right ear lobe

Standardized values of the last two columns were calculated by dividing the values of the first two columns by the standard deviation of the middle column. Standardized values allow comparison between methods of measurement. A standardized mean of the differences close to zero indicates that data can be used to examine repeatability (i.e. knowledge of the first measurement is unlikely to alter the second measurement). A smaller standardized repeatability coefficient indicates a better repeatability (e.g. ears better than fingers). According to the British Standards Institution, it should be below 1 www.bsigroup.com.

## 4. Discussion

The information on pain sensitivity provided by a calibrated clothes peg versus the measurements with an established electronic algometer (Somedic Type II) correlate at a clinically meaningful level, virtually all revealing large effect sizes according to Cohen's conventions. The clothes peg values show this correlation both in respect to the *pressure pain detection threshold *(PPdt) and the *pressure pain tolerance threshold *(PPtt). This meaningful level of correlation could equally be seen in both anatomical sites tested (i.e., middle finger and ear lobe) as well as in orthopaedic and non-orthopaedic patients. Moreover, the validity check for repeatability yielded comparably stable and highly reproducible values. All of these correlations and qualitative similarities suggest that the two methods may be to some extent interchangeable.

Interestingly, the correlation between the two compared algometric methods was achieved even though the detailed measurement approach is not exactly identical for the two methods. Specifically, the electronic pressure algometer checks for the *PPdt*, at which, in response to gradually increased pressure applied, *non-painful *perception of pressure changes into *painful *perception of pressure. At the onset of pain, the test stops at once. In contrast, the pressure applied with a clothes peg is invariable. Patients tend to perceive the steady amount of pressure on their *finger *as being *beneath or slightly above *the pressure pain threshold. The pressure exerted by a clothes peg on an ear lobe, however, is perceived as being consistently and clearly *above *the pain threshold. Therefore, the clothes peg test on the ear indirectly assesses the patient's ability to *endure pain*. Electronic testing using a pressure algometer checks for a patient's *ability to endure pain *if *PPtt *is determined. Therefore, clothes peg exposure tests integrate aspects of pain sensitivity, which are otherwise accounted for separately by assessing PPdt and PPtt, and they correlate with both parameters to the same extent.

From a methodological point of view, it might be considered as a disadvantage that the clothes peg test does not explicitly distinguish between PPdt and PPtt. Therefore, in a clinical setting it seems recommendable to consistently apply the clothes peg method to both anatomical sites (i.e. finger plus ear lobe) as they *differ in pain sensitivity*. Even if average values of the clothes peg test obtained on ear lobe and finger are highly correlated, individual patient testing often reveals variations in reaction to patterns depending on whether the clothes peg is applied on a finger (pressure sensitivity in proximity of the pain threshold) or on an ear lobe (endurance of pressure pain close to the tolerance limit). It is further worthwhile to carry out the clothes peg test symmetrically on both sides of the body. This sometimes reveals differences in perception between body sides that are not uncommon in chronic pain patients and may point to pain-associated nondermatomal somatosensory deficits (NDSDs) [10].

There are no other studies on clothes peg algometry as a method of measuring pain. Therefore, there are currently no published data available with respect to the discriminatory performance of the clothes peg algometer in different forms of clinical pain entities reaching beyond those investigated in the present study. The observed difference in pain sensitivity between the orthopaedic and medical-psychosomatic pain patients is, however, suggestive for discriminatory diagnostic properties (Table 2). Further investigations are needed to see whether clothes peg algometry is a useful clinical tool for discriminatory purposes in pain patients [12]. Furthermore, data on *normal values *in the pain-free population are useful in order to have reference values. Such reference values have been recently become available for a variety of psychophysical and electrophysiological pain tests, including the electronic pressure algometer we used [13,14]. Furthermore, we also generated first reference values for the clothes peg algometer [15].

## 5. Conclusion

To conclude, clothes peg algometry is both an easy-to-administrate and low-cost method for assessing patients' pain sensitivity where electronic pressure algometers are not readily available. Clothes pegs can easily be stowed away in a jacket pocket, they are ubiquitous worldwide and their clamping forces can easily be checked (Figure 1). This simple and inexpensive method to test pain sensitivity deserves further investigation, since it has the potential for becoming widely used in clinical practice.

## Competing interests

The authors declare that they have no competing interests.

## Authors' contributions

NE and MLG are responsible for the whole clinical concept and examinations, and RvK and RC for the study design and the statistical analyses. MC supervised the study on a medical-methodological level. NK, BW, and EM performed the clinical tests. All authors participated substantially in the acquisition or the analyses of the data. All co-authors were involved in drafting or revising the manuscript and have approved its final version.

## Pre-publication history

The pre-publication history for this paper can be accessed here:

http://www.biomedcentral.com/1471-2474/12/174/prepub
